# Experiences and preferences of care among Swedish immigrants following a prenatal diagnosis of congenital heart defect in the fetus: a qualitative interview study

**DOI:** 10.1186/s12884-016-0912-1

**Published:** 2016-06-02

**Authors:** Tommy Carlsson, Ulla Melander Marttala, Elisabet Mattsson, Anders Ringnér

**Affiliations:** Department of Public Health and Caring Sciences, Uppsala University, BMC Husargatan 3, Box 564, S-75122 Uppsala, Sweden; Department of Scandinavian Languages, Uppsala University, Uppsala, Sweden; Department of Health Care Sciences, Ersta Sköndal University College, Stockholm, Sweden; Department of Nursing, Umeå University, Umeå, Sweden

**Keywords:** Immigrants, Interview, Life change events, Pregnancy, Prenatal diagnosis

## Abstract

**Background:**

Immigrants experience significant challenges when in contact with healthcare and report less satisfaction with maternity care compared to native Swedes. Research that gives voice to pregnant immigrant women and their partners following a prenatal diagnosis of a fetal anomaly is scarce. Thus, the aim of this study was to explore experiences and preferences of care following a prenatal diagnosis of congenital heart defect among Swedish immigrants.

**Methods:**

Pregnant immigrants and their partners were consecutively recruited following a prenatal diagnosis of a congenital heart defect in the fetus. Nine respondents were interviewed in five interviews, four with the aid of a professional interpreter. The material was analyzed using manifest qualitative content analysis.

**Results:**

The analysis resulted in five categories: 1) “Trustworthy information”, 2) “Language barriers”, 3) “Psychosocial situation”, 4) “Peer support”, and 5) “Religious positions”.

**Conclusion:**

The potential need for interpreter services, visual information, psychosocial support, coordination with welfare officers, and respect for religious positions about termination of pregnancy are all important aspects for health professionals to consider when consulting immigrants faced with a prenatal diagnosis of fetal anomaly in the fetus. Peer support within this context needs to be further explored in future studies.

## Background

The United Nations Educational, Scientific and Cultural Organization (UNESCO) defines the term migrant as “any person who lives temporarily or permanently in a country where he or she was not born, and has acquired some significant social ties to this country” [1]. In December 2014, Sweden had a population of approximately 9.7 million, of whom 700,000 were foreign citizens and 937,016 Swedish citizens with a foreign country of birth. In recent years, Swedish immigration has increased and in 2014 approximately 127,000 individuals immigrated to Sweden [2]. Immigrant pregnant women attend fewer antenatal care appointments [3] and face a number of specific challenges when in contact with health professionals, for example a lack of familiarity with western healthcare systems [4, 5], language barriers [4–6], health literacy difficulties [6], and possible history of severe trauma [5]. These challenges are further increased by cultural barriers and economic obstacles to reaching health information [6]. Immigrant women suffer from worse pregnancy outcomes in comparison to native women, including risk of low birth weight, perinatal mortality [3, 5], preterm delivery, and congenital malformations, especially in countries with weak integration policies [7]. Pregnant women desire individualized care and adequate information during their pregnancy. However, immigrant women report less satisfaction with maternity care compared to native women [4] and experience miscommunication in settings of maternity care [8].

The detection rate of congenital heart defects (CHD) in the fetus has greatly increased since the introduction of routine ultrasound screening of pregnant women [9]. Pregnant women have optimistic expectations of the routine ultrasound screening [10–12], resulting in unpreparedness for a prenatal diagnosis of a fetal anomaly [10, 11, 13, 14]. A prenatal diagnosis involves acute grief reactions [15] and psychological distress [15–17]. Furthermore, depending on current laws, termination of pregnancy may be an option. The decisional process to continue or terminate the pregnancy involves ethical considerations [18] and informational difficulties [19, 20]. Previous studies have observed that religious positions have an impact on reasoning about termination of pregnancy if a fetal anomaly is detected during prenatal screening procedures [21, 22], and that maternal ethnic origin is associated with the decision to terminate the pregnancy following a prenatal diagnosis of CHD [23, 24].

According to Kreps and Sparks [6], development of policies and guidelines concerning health information to immigrants is a complex process that needs to be evidence-based and involve careful strategic planning. Thus, to gain an understanding of how care should to be planned and provided in order to reach equity between immigrant and native pregnant women following a prenatal diagnosis, experiences and preferences of care should be explored in both groups. However, as far as we know, inductive qualitative research within the field of prenatal diagnosis is scarce regarding immigrants. Thus, the aim of this study was to explore experiences and preferences of care following a prenatal diagnosis of a congenital heart defect in the fetus among Swedish immigrants that decided to continue the pregnancy.

## Methods

### Study design

This study was an explorative study that used qualitative methods to gain insight into and describe a certain phenomenon [25], i.e., the experiences following a prenatal diagnosis of a major CHD.

### Study context

This study was conducted at the tertiary referral center for fetal cardiology and fetal medicine at Uppsala University Hospital, Sweden. In Sweden, all pregnant women are offered a routine ultrasound screening at approximately 16-18 weeks of gestation. If a CHD is suspected during this examination, a referral is made for a consultation by a fetal cardiologist. Based on the findings and precision of the diagnosis, information about a broad variety of topics is provided by the fetal cardiologist. Typically, the specialist also provides an illustration of the CHD in addition to oral information. When necessary, interpreter services are used. Additional fetal medicine investigations are also routinely offered. According to the Swedish abortion act, pregnant women are entitled to terminate the pregnancy up until gestational age 18 weeks and 0 days and afterwards apply for approval for termination of pregnancy from the National Board of Health and Welfare. If the pregnant woman chooses to continue the pregnancy, follow-up visits are offered every 4-6 weeks to monitor the progression of the CHD and prepare for the delivery, in addition to the fetal medicine program.

### Participant recruitment

The participants were consecutively recruited as they attended a specialist follow-up consultation visit in the unit for fetal cardiology and fetal medicine, Uppsala University Hospital, following a decision to continue the pregnancy. To be considered for inclusion, potential participants needed to be immigrants, and pregnant/partner to a pregnant woman with a fetus diagnosed with a major CHD, i.e. in need of corrective surgery within one year after birth. Elisabet Mattsson, a specialist nurse and midwife at the unit, was responsible for the recruitment which took place in a set time period between September 2014 and April 2015. In total, five pregnant women and their four male partners were asked to participate and all consented to be interviewed.

### Characteristics of the study sample

The mean age was 33 years (range = 22-43) and the mean time of living in Sweden was 7 years (range = 1-17). The mean gestational age at diagnosis was 22 weeks (range = 19-32). Seven of the participants had used interpreter services during the specialist consultation at the time of diagnosis and two participants (one couple) had declined interpreter services. The prenatal diagnoses included single ventricle (*n* = 4, 45 %), transposition of the great arteries (*n* = 3, 33 %), and ventricular septal defect (*n* = 2, 22 %). Table 1 presents characteristics of the sample.

**Table 1:** Characteristics of the sample (*n* = 9)

Characteristic	Category	*n*(%)
Country of birth	Iraq	4(45)
	Somalia	3(33)
	Palestine	2(22)
Born children	1	6(67)
	3	2(22)
	>3	1(11)
Highest education	None	2(22)
	Compulsory education	2(22)
	Senior high school	2(22)
	College/University	1(11)
	Not disclosed	2(22)
Sex	Female	5(55)
	Male	4(45)

### Data collection

The participants were given the option to either be interviewed together with their partner, or individually. One woman was single and was thus interviewed individually and all of the couples chose joint interviews. The second author, a female sociolinguistic researcher with previous experience of conducting face-to-face interviews and with no clinical contact with the participants, conducted all five interviews in Swedish. A professional interpreter was used in four of the five interviews, which lasted 25 to 45 min (Mean = 35) and took place at Uppsala University Hospital. One couple chose not to use an interpreter during the interview, as they felt that they had sufficient knowledge of Swedish. The interviews were digitally recorded and transcribed verbatim. However, two interviews with four respondents were not recorded because of respondent wishes. Handwritten notes were kept during all interviews. An interview guide was used to stay relevant to the aim while allowing for flexibility [25]. Table 2 presents the interview guide.

**Table 2:** Interview guide

Main question	Sub-questions
1. Can you tell me a bit about your appointment today?	What was done?
	What was said?
2. How do things feel today?	
3. What plans are there for the time ahead?	
4. Can you tell me about what has happened?	
5. What are your thoughts, experiences?	How do things look now?
	How do your thoughts go now?
6. How were you told about it?	What went through your mind?
	What did you do?
7. Have you thought a lot about what you should do?	What is the most difficult thing?
8. What information have you been given?	Who gave you the information?
	How were you given the information?
	What sort of information did you get?
	What was good?
	What was missing?
	Was the information easy or difficult to understand?
	What did you want to ask about?
9. What other people outside the hospital did you talk to?	How was that?
10. What different kinds of information did you get and search for yourselves?	The Internet: Any special websites and what do you say about them?
	What information feels trustworthy and why? How do you assess it?
11. How did you find out about different things?	
12. Would a special website be a good idea?	What should it contain?
	What would it look like?
13. Anything else that you’ve thought about that you would like to say?	

### Analysis

To check for interpreter bias during the interview, one interview transcript including interpreter wording was subjected to a complete translation by a professional translator. The translated material revealed only small differences of limited importance and the work of the interpreter was considered sufficient and adequate. The first author, a male PhD-student and specialist nurse with training and previous experience of qualitative content analysis, was responsible for the primary analysis which was conducted utilizing Nvivo for Mac version 10.2.0 (QSR International, Doncaster, Victoria, Australia). Initially, the interviews were listened to and the transcripts read several times to gain a sense of the whole. Subsequently, the handwritten notes and interview transcripts were subjected to qualitative content analysis, a method for systematically describing the content of any form of communication [26]. Meaning units, defined as words/sentences/paragraphs containing aspects related to each other through their content and context, were identified, condensed, and assigned a descriptive code. These codes were structured into categories that shared a common subject and described the manifest content, i.e., the visible and obvious content. The first and second author held discussions during the primary analysis to ensure consensus. When the primary analysis was considered complete, all authors held joint discussions until consensus was reached.

### Ethics statement

Ethical approval to conduct the study was obtained from the Regional Ethical Review Board in Uppsala, Sweden (Approval number: 2014/504). With the aid of professional interpreters, written and verbal information was given to prospective participants, and written consent was collected before enrollment. Professional interpreters were present during the interviews, with the exception of one interview with a couple.

## Results

The analysis resulted in five categories: “Trustworthy information”, “Language barriers”, “Psychosocial situation”, “Peer support” and “Religious positions”. The categories will be presented in the following sections.

1. **Trustworthy information**

Trustworthy information was highly valued following the diagnosis, especially from the specialist cardiologist, which was described as irreplaceable medical information from a reliable expert and the best source of information. The respondents were all content with the information received by the health professionals and considered it sufficient.

*He explained really well actually and we also got, he also drew on piece of paper what he was going to do and what is wrong with her.* (Female 2)

While the respondents had been satisfied with the information provided by the specialist physician, some also described different strategies to find supplemental trustworthy information. Some used the Internet to search for information and appreciated the information found online. Others, on the other hand, had chosen not to use the Internet for supplemental information following the diagnosis. Reasons for this included the fact that the information from the specialist had been enough, that the online information would raise fear about the future, lack of specific and reliable information, and difficulties to interpret the information found online. Also, a fatalistic trust in God was stated as a reason not to search for more information:

*Yes but so we believe that our entire destiny comes from God, so if you read something that it might happen, then it will turn out to be wrong. And something can happen that will hurt you. So you forget about it and believe in God and so on. There is the Internet and computers but we don’t use it for this.* (Female 1)

Some respondents who had used the Internet for supplemental information described that while they had found information in English and their mother tongue, less had been found in Swedish. When asked about the value of a supplemental information website produced and delivered by health professionals, all respondents were optimistic and thought that it would have been valuable to them. According to the preferences of the respondents, this website should contain trustworthy information from health professionals, provided in the respondents’ mother tongue.

*[Interviewer: A special website run by the hospital, what do you think, would it be a good idea?] Great, it would be great, you much more information in Swedish because there isn’t so much in Swedish in fact.* (Female 2)

2) **Language barriers**

The respondents described that medical terminology had been a barrier to comprehension of information, and this needed to be respected by the cardiologist during the consultation. The words had been easier to grasp in their mother tongue.

*There are a lot of concepts that we don’t understand which the doctor uses […] yes what the doctors in the Arabic language also our country that we understand these concepts better we studied in the Arabic language. And we know them in Arabic.* (Female 2)

One way to overcome language barriers seemed to be use of visual information. All respondents had appreciated that the specialist drew an illustration of a normal heart and the heart defect, which had deepened their understanding of the defect. The same was described about information found online, for example respondents had viewed videos on the Internet to receive visual information, supplemented by a translation into English.

*I tried to watch a video that was in English, which showed how the disease comes about and so on, I understood when they showed pictures, but then you can get an English translation.* (Female 3)

3) **Psychosocial situation**

Psychological distress was described following the initial detection, which was made worse by the delay between the routine ultrasound scan and the specialist consultation. The respondents worried about both the intrauterine status of the fetus and the postnatal situation of their child and family. Thus, questions and concerns about the postnatal care of their prospective child were expressed. Follow-up visits to check the status of the fetus were a valued way of easing worry during the remaining part of the pregnancy.

*At first we were tired and mentally exhausted, in bad mental shape at the beginning.* (Male 1)

Some respondents described living in a vulnerable social situation, which impacted the situation with their prospective child. For example, one respondent who was single described significant issues with her small apartment that she shared with her many children. This caused worry about the future postnatal situation, as a child with CHD will need special care. To solve this issue, she wanted a doctor’s certificate for the landlord so that she would be able to move to a larger apartment.

*And I have a little problem, that is where I live now. I’ve got [many children] and pregnant, one on the way and he’s not in good shape, not good… And I have a one-bedroom flat, that’s the… That is, it’s a very small home, the flat that is, so it’s a bit of a problem. It’s very difficult when the child has come, the baby has come and where will we live like…* (Female 1)

4) **Peer support**

In addition to information from the specialist, the respondents also came in contact with lay persons who were parents to children with CHD. These parents had been encountered via their social networks and were thus linguistically and ethnically concordant with the respondents. Meeting these persons had been comforting, as they felt calmer after seeing for themselves that children with CHD can have healthy and joyful lives. Information from parents was valued in different ways than information from health professionals, as parents could give advice on how to care for the child and family.

*[Information] about the heart, the heart defect [it is] always better information if [it is from] a doctor, because they can explain in a good way, so to speak. But [meeting] families that is [an] other thing, yes, afterwards they so to speak what [you] should do [with the child].* (Male 2)

Some respondents also expressed that an internet-based information platform about CHD would enable parents to get in touch with each other and provide opportunities to meet and discuss with other parents to children with CHD.

5) **Religious positions**

The respondents described that their fate was in the hands of God and that what had happened was the will of God. They expressed thankfulness to God regardless of their present and future situation.

*Nothing wrong, we are grateful, we are great believers, what happens, it happens, from God’s side.* (Female 1)

This was also reflected in their decision to continue the pregnancy, as their faith would not allow termination of pregnancy. Because of their religious positions, some respondents questioned the reasons behind information about termination of pregnancy. Thus, they preferred information about termination of pregnancy that is adapted to their religious positions.

*We could take this shocking information [about abortion] but we don’t know how the other families could react. One thing we must add, we are Muslims. Having an abortion, it’s not allowed.* (Male 1)

## Discussion

The findings reflect that health professionals need to provide trustworthy information and take into account language barriers, potential vulnerable psychosocial situations, and religious positions regarding pregnancy termination when counselling immigrants faced with a prenatal diagnosis of a major CHD. Furthermore, translating services, peer support, and supplemental visual information are tools to promote comprehension of information and lessen the negative effects of potential language barriers.

Low health literacy, i.e., the degree to which individuals have the capacity to obtain, process, and understand health information [27], is associated with poor health outcomes [28] and limited understanding of medical information [29, 30]. We cannot conclude that the respondents in our study had low health literacy, but they experienced comprehension difficulties during the specialist consultation, specifically with regard to medical terminology. It has been reported that the specialist consultation may include difficult terminology [20], and difficulties understanding medical terminology may be increased among immigrants. Health professionals need to be mindful of these difficulties when consulting non-native pregnant women and partners following a prenatal diagnosis and offer translating services and comprehensive explanations when needed.

The Internet was used by some of the respondents to find supplemental information. It has been concluded that supplemental written information promotes knowledge and satisfaction [31], and is desired following a prenatal diagnosis [11, 12, 19, 20]. Tailored communication technologies have been proposed to support health education among immigrants [6] and following a prenatal diagnosis [12, 20]. While some of the respondents did not use the Internet to search for supplemental information, all were optimistic about a specific website with trustworthy information delivered by health professionals. Thus, online supplemental written information in specific languages for immigrants faced with a prenatal diagnosis of a fetal anomaly is an important step to reduce inequalities in health care due to different ethnic backgrounds. Consequently, immigrants need to be involved in future planning and testing of Internet-delivered information platforms.

In line with previous research in prenatal settings [11, 20], the respondents expressed particular appreciation of visual supplemental information, such as illustrations of the CHD and online videos. Illustrations have been found to markedly improve attention and recall of information and are particularly beneficial for individuals with low health literacy [32], and they are appreciated by immigrants [33]. A study of an online experiment comparing different modalities of information among individuals with high and low health literacy reports that animations combined with spoken language significantly improves information recall and results in a similar amount of information recall regardless of high or low health literacy levels [34]. Thus, it can be theorized that videos and animations with spoken language in the mother tongue are the most suitable modality when providing supplemental information to immigrants following a prenatal diagnosis. The fact that visual information is valued needs to be integrated into clinical practice when consulting immigrants following a prenatal diagnosis, and needs to be tested in future experimental studies.

It is widely recognized that a prenatal diagnosis results in psychological distress [15–17] and acute grief reactions [15]. Similarly, the respondents in this study described psychological distress following the initial detection. However, for some of the respondents the distress was increased by a vulnerable social situation, for example small living quarters with a large family. In 2014, 30% of Swedish immigrants from a country outside of Europe reported that they lacked emotional support, and 18% that they lacked practical support [35]. Thus, health professionals need to be attentive to the social situation and needs of immigrants faced with a prenatal diagnosis, and apply a team-based approach by involving professional psychosocial support when necessary. Judging from the findings of this study, immigrants seek peer support by establishing contact with linguistically and ethnically concordant parents to children with CHD. It is possible that health professionals could promote emotional support by establishing a network of contact persons with similar experiences. However, the impact of such support needs to be investigated in future studies.

We also observed that the respondents put their fate in the hands of God following the diagnosis. Similar findings have previously been reported among Somali immigrants in British obstetric settings [8] and for antenatal screening decisions among Turkish immigrants in the Netherlands [21]. Considering the associated existential and ethical issues of life and death [18], it is possible that values of fatalism are especially present following a prenatal diagnosis. Awareness of religious and ethical values is essential among health professionals counseling individuals faced with a prenatal diagnosis. In the case of Islam, even though termination of pregnancy following a prenatal diagnosis of severe fetal anomalies is allowed according to a Fatwa, i.e., a legal interpretation of the Qur’an or other Islamic law [36], opposite views have also been described [21]. Through the findings from this and another study [37], we can conclude that clinicians should explicitly ask about religiosity and what consequences it may have in this situation rather than assuming either a certain set of values based on ethnic origin or one’s own ethical stance. However, in a Swedish context physicians are obligated by law to inform patients about the option to terminate the pregnancy. Thus, health professionals should be mindful of both religious positions and abortion laws when consulting about termination of pregnancy and deliver information accordingly.

### Methodological considerations

This study aimed to explore experiences and needs among Swedish immigrants following a prenatal diagnosis of CHD. Thus, qualitative methods were chosen to inductively explore lived experiences. In total, nine respondents were interviewed, included through clinical consecutive recruitment during eight months. While we recognize that the sample size is small, this study is to our knowledge the first to inductively explore experiences among immigrants faced with a prenatal diagnosis. Thus, the findings should be considered hypothesis generating and warrant further research within this population.

In order to validate the translations from the professional interpreters, one random transcript was subjected to an additional translation from a different professional interpreter. The result did not reveal any substantial loss of content due to interpreter services. Thus, we consider it unlikely that the results in this study are affected by interpreter bias. For two of the interviews, participants declined the use of a recording device. In these cases, the interviewer kept handwritten notes. Furthermore, the interviewer had an active part in the data analysis.

Only cases that had chosen to continue the pregnancy were recruited in this study and thus they do not reflect experiences among those terminating the pregnancy. Furthermore, we acknowledge the fact that the majority of the populations within all of the included countries are Muslims. We did not ask specific questions concerning respondents’ religious beliefs or reasons for immigration, such as potential refugee backgrounds. No respondents mentioned a background as a refugee and all had Swedish residence permits at the time of the interview. Taken together, it is possible that the findings are not transferable to refugees or to persons with specific religions, particularly considering the findings regarding religion and termination of pregnancy. However, the fact that the respondents were consecutively recruited during several months, and that all of the included countries of birth are represented among the ten most common countries of birth among Swedish immigrants in 2014 [38], strengthens the transferability to Swedish settings. To increase the trustworthiness of this study, both the first and the second author listened to all recordings and read all transcripts. Further, all authors took part in compiling the final results.

Participants in this study had the option to choose individual interviews, but all couples preferred to be interviewed together. Joint interviewing has the advantage of being generally accepted by participants and may enhance the interview atmosphere by offering a more comfortable situation for some [39]. In addition, the method is likely to improve participation [40], an important aspect to consider when studying relatively rare phenomenon. Methodological disadvantages of joint interviewing mostly concern the interaction between the two participants [41]. In our study, it could be speculated that the interaction between the couples led to relatively short interviews (mean 35 min). Judging from our data, both men and women shared their experiences, except for one woman who was rather quiet. It is impossible to know if this woman would have talked more or less without her partner present during the interview, or if she would have declined participation in an individual interview. It has been suggested that joint interviews are a particularly appropriate method for studying complex shared practices [42] such as experiences and preferences of care following a prenatal diagnosis of a fetal anomaly. Consequently, we consider the findings credible and worth taking into account in clinical practice.

### New contribution to the literature

This study contributes to the scarce literature concerning experiences of immigrants that decide to continue the pregnancy following a prenatal diagnosis of CHD. In line with previous studies, psychological distress [15–17] and informational difficulties [19, 20] were observed. In addition, Swedish immigrants describe a desire for peer support from linguistically and ethnically concordant parents to children with CHD. They also experience specific difficulties, i.e., language barriers, vulnerable social situations and a healthcare system that informs about pregnancy termination without taking into account religious positions.

## Conclusion

The potential need for interpreter services, visual information, psychosocial support, coordination with welfare officers, and respect for religious positions about termination of pregnancy are all important aspects for health professionals to consider when consulting immigrants faced with a prenatal diagnosis of fetal anomaly in the fetus. Peer support within this context needs to be further explored in future studies.

## Abbreviations

CHD congenital heart defects
